# Reproductive potential of fall armyworm *Spodoptera frugiperda* (J.E. Smith) and effects of feeding on diverse maize genotypes under artificial infestation

**DOI:** 10.3389/finsc.2022.950815

**Published:** 2022-09-05

**Authors:** Geoffrey N. Anyanda, Anani Y. Bruce, Dan Makumbi, Monday Ahonsi, Ruth Kahuthia-Gathu, Samita E. Namikoye, Yoseph Beyene, B. M. Prasanna

**Affiliations:** ^1^ Global Maize Program (GMP), International Maize and Wheat Improvement Centre (CIMMYT), Nairobi, Kenya; ^2^ Department of Agriculture Science and Technology, Kenyatta University, Nairobi, Kenya; ^3^ Amobant LLC, Fort Wayne, IN, United States

**Keywords:** maize hybrids, fall armyworm, life table, yield loss, artificial infestation

## Abstract

Fall armyworm (FAW) *Spodoptera frugiperda* (J.E. Smith) has become a major threat to maize production in Africa. In this study, six maize genotypes were assessed for their resistance to FAW under artificial infestation in both laboratory and net house conditions. These included two FAW-tolerant hybrids (CKHFAW180294 and CKH191221), two commercial hybrids (WE2115 and CKH10717), and two open-pollinated varieties (ZM523 and KDV4). Larval development time and reproductive potential were assessed on maize leaves in the laboratory and a life table for FAW was constructed. The maize genotypes were also artificially infested with three FAW neonates at two phenological stages (V5 and V7) and reproductive stage (R1) in the net house. Leaf and ear damage scores were recorded on a scale of 1–9. Larval development time varied significantly between maize genotypes with the highest on CKH191221 (16.4 days) and the lowest on KDV4 (13.7 days). The intrinsic rate of natural increase for life tables varied from 0.24 on CKH191221 to 0.41 on KDV4. Mean generation time of FAW ranged from 17.6 to 22.8 days on KDV4 and CKH191221, respectively. Foliar damage was the lowest on CKH191221, and the highest on KDV4 at V7 infestation stage in week 1. CKH191221 had the lowest ear damage score, whereas ZM523 had the highest scores at V5 infestation stage. The highest and lowest yield reductions were observed on ZM523 (64%) at V7 infestation stage and CKHFAW180294 (6%) at R1 infestation stage, respectively. The results indicated the potential for developing tropical mid-altitude maize germplasm with native genetic resistance to FAW.

## Introduction

Maize (*Zea mays* L.) is one of the most important staple food crops in Sub-Saharan Africa (SSA), contributing to the food security and livelihoods of millions of smallholder farmers (1). In the last 6 years, maize production in SSA has been constrained by the invasion of fall armyworm (FAW), *Spodoptera frugiperda* (J.E. Smith) (Lepidoptera: Noctuidae). This pest, native to the Americas, was first formally reported in Africa in 2016 (2) and, since then, has caused substantial maize yield losses (3–6). In SSA, FAW encountered suitable ecological conditions and quickly became one of the most damaging pests of maize within 2 years of arrival on the continent (7). The FAW larvae feed on young whorls, ears, and tassels of maize plants (8). Since 2016, an array of management techniques was tested, validated, and deployed in Africa. These methods include agroecological control that applies knowledge about the complex interactions and the environment, e.g., push–pull technology and cultural control such as crop rotation and intercropping. The biological control using organisms or their components has achieved an efficient strategy for managing the pest such as adoption of natural enemies, such as *Trichogramma* spp. and *Telenomus* spp., as well as the biorational control using botanicals and biopesticides, such as nucleopolyhedroviruses. Host plant resistance (HPR) including transgenic expressing *Bacillus thuringiensis* (Bt) toxins can also be used to control FAW and chemical pesticide; many of these products are too expensive and inaccessible to be considered by many smallholder farmers in SSA. Each of these management options has limitations when used alone. An integrated approach is the best options for sustainability in controlling FAW in Africa (7, 9).

HPR is a control strategy that focuses on identification and deployment of germplasm with native genetic resistance to FAW. It is a key component of any pest management strategy (7, 9–11). There is a need to develop and deploy insect-resistant crop varieties as a main component of an Integrated Pest Management strategy against FAW in Africa and Asia (7, 9–12). The quantitative or polygenic nature of native genetic resistance to FAW offers the opportunity to minimize selection pressure on the pest and prevents emergence of new resistant strains. Resistance to insect pests is the ability to minimize feeding damage through mechanisms/types such as antixenosis, antibiosis, and tolerance (13). “Antixenosis” denotes presence of morphological or chemical factors that alter insect behavior, resulting in poor establishment of the insect pests (poor feeding, rejection of oviposition, and delay acceptance as host). “Antibiosis” is the effect of the host plant on the biology (low survival rate, longer development period, and low adult fecundity due to biochemical factors). “Tolerance” is the capacity of the plant to withstand insect attack and give optimum yields despite the insect damage (with minimal yield penalty).

The International Maize and Wheat Improvement Center (CIMMYT) has engaged in developing germplasm with insect resistance since the 1980s, and multiple borer-resistant (MBR) and multiple insect-resistant tropical (MIRT) inbred lines and populations were developed. With the outbreak of FAW on the African continent, CIMMYT started an extensive effort to screen under artificial infestation large sets of germplasm [inbred lines, hybrids, improved open-pollinated varieties (OPVs), and populations such as MBR and MIRT] from earlier breeding efforts at CIMMYT and initiatives like the more recent Insect Resistant Maize for Africa (9–12).

From 2017 to 2019, over 3,300 maize genotypes were tested under FAW artificial infestation at Kiboko. This preliminary screening led to the identification of several inbred lines and OPVs with tolerance and/or resistance to FAW. Some of the inbred lines identified were used to form more than 2,030 hybrids that were also tested under FAW artificial infestation at Kiboko. Two promising hybrids were identified and considered tolerant due to their lower leaf, ear damage, and higher grain yield under FAW artificial infestation. However, no detailed studies have been undertaken to investigate the basis of tolerance and/or resistance to FAW in this germplasm. This study therefore investigated different parameters to explain the basis of tolerance or resistance in a set of genotypes. The bionomic parameters observed include the pre-imaginal developmental time, larval and pupal weight, adult longevity, fecundity, sex ratio, and emergence rate to FAW on six selected maize genotypes under laboratory conditions. The extent of damage caused by FAW on the same set of genotypes under FAW artificial infestation in net house conditions was also evaluated.

## Materials and methods

### Plant material

Six maize genotypes were selected for this study. These included two FAW-tolerant experimental hybrids (CKH191221 and CKHFAW180294), two commercial hybrid checks (WE2115 and CKH10717), and two popular commercial OPVs (ZM523 and KDV4). All the genotypes were developed at CIMMYT using different source germplasm selected for various attributes including high yield potential, drought tolerance, early/intermediate maturity, and tolerance to stem borers. CKH191221 and CKHFAW180294 were developed in 2018, and their parents have a background of insect-pest resistance. WE2115 and CKH10717 are drought-tolerant hybrids that are commercially grown in Kenya and Tanzania, respectively. ZM523 is a drought-tolerant intermediate maturity OPV that is commercially grown in several countries across SSA, whereas KDV4 is an early maturing OPV that is extensively commercialized in Kenya. These OPVs were developed from multiple stress-tolerant inbred lines.

### Insect culture

A colony of FAW was established during the long rain season of 2017 by collecting 100 larvae and 10 pupae at Kiboko (2°15′S 37.75′E, 975 masl) and 50 larvae and 10 pupae at Machakos (1°31′S, 37°16′E) in Kenya. The colony was maintained in the insectary at Kenya Agricultural and Livestock Research Organization (KALRO) at Katumani (1°35′S, 37°15′E, 1,610 masl) on artificial diet as described by Prasanna et al. (10). In brief, an artist brush was used to place 30 neonates in a jar (12 × 14.5 × 11.5 cm). At the third instar, one larva was placed on each diet vial (2.5 × 8.5 × 2.5 cm) until pupation. The pupae were placed in a petri dish in the oviposition cage until emergence to allow the pairing and egg laying. The FAW colony is maintained under controlled conditions at 27 ± 1°C; 12:12 h (light, dark) and 70 ± 5% RH (10).

### Analysis of reproductive potential of FAW

Life table studies for FAW were conducted at the KALRO-Katumani Insectary in 2019. Three maize seeds of each genotype were planted per pot measuring 15 cm diameter and 30 cm height. A total of 30 pots for each maize genotype were established in a screenhouse. Soil obtained from Katumani (Ferrosol) with dry manure at a ratio 3:1 (soil: manure) was used. Maize leaves from V5 to V10 were used as natural diet for rearing the FAW larvae. A single freshly emerged neonate was picked using a fine soft camel hairbrush and placed in a 25-ml plastic vial with screw cap (with pinholes to allow air circulation and prevent escape of neonates or larvae) measuring 2.5 × 6 cm with two pieces of fresh leaves (3.0 cm each) from each of the test genotypes. A piece of napkin was placed in the vial to absorb moisture. The vials were then kept in an insect rearing laboratory room with controlled conditions (27 ± 1°C; 12:12 h (light, dark), and 70 ± 5% RH). The neonates were then observed daily for mortality until pupation. The diet was changed after every 2 days to allow ample feeding time. Weight (mg) of the larvae was taken after 6 and 9 days using a Shimadzu electronic balance (Type ATY224 AP, Japan). The duration of larval and pupal development period was recorded. The pupae were kept under the same conditions as larvae until adult emergence. The experiment was arranged in a randomized complete block design with three replications (insect cohorts) and 80 insects (vials) in each replication (n = 3 × 80 = 240).

The newly emerged naïve adults (without oviposition experience) were paired using moths reared from the same maize genotype for mating and egg laying. Three leaf pieces (3–5 cm) from the respective maize genotypes were placed in a plastic container measuring 11 × 15 × 10 cm where moth pairs were introduced for mating and oviposition. The container was covered with a perforated lid for ventilation. The moths were fed on a 10% sugar solution soaked in cotton wool. After every 24 h, the leaf pieces were changed, and the eggs laid counted under a dissecting microscope (type Optika Vision Lite 2.13) and recorded. This procedure was repeated until the female moths died. The eggs laid on the leaf pieces of the different maize genotypes were placed in individual petri dishes for hatching. The number of neonates that emerged after hatching was counted to determine the viability of the eggs.

### FAW artificial infestation in net house

FAW artificial infestation trial was conducted at the FAW Screening Facility at Kiboko, Kenya, in 2019. The mean temperature at Kiboko ranges from 16.5°C to 28.6°C. The rainfall pattern is bimodal with sometimes unpredictable short rains between March and April and long rains from October to December. The average precipitation is 545–629 mm per year. The soils are classified as Acri-Rhodic Ferrassol. The soil obtained from Kiboko (Ferrasols to luvisols) with dry manure at a ratio 5:1 (soil manure) was used. Fertilizer application was done at the rate of 60 kg of nitrogen and 60 kg of phosphorus (P_2_O_5_) per hectare. Manual weeding was carried out when necessary to keep the plots clean. The plants were regularly irrigated.

The trial was set up as a split plot design with three replications at Kiboko, Kenya, in insect-proof screenhouses (gauges 0.4 × 0.9 mm) measuring 1,000 m^2^, divided into four compartments, each measuring approximately 250 m^2^. The main plot treatments were three phenological stages: V5 (five leaves fully emerged), V7 (seven leaves fully emerged), R1 (silking), and the control (compartment without FAW infestation). The subplots were the six maize genotypes. Each subplot consisted of nine rows measuring 3 × 6.75 m, planted at spacing of 0.25 × 0.75 m with a space of 1 m between the subplots giving a total of 324 plants per subplot. Two seeds were sown per hill, and thinning was done at 2 weeks after planting leaving one plant per hill. Maize genotypes were artificially infested with three FAW neonates per plant at each of the three phenological stages (V5, V7, and R1) following the procedure described by Prasanna et al. (10). The infestation done at V5 and V7 phenological stages had four generations of insects before harvesting, whereas the one done at R1 phenological stage had two generations of insects before harvesting. Artificial infestation was done between 4 p.m. and 6 p.m. to allow the neonates to acclimatize to the environment before the following day changes in temperatures and relative humidity that may otherwise desiccate the neonates. An insect-proof screenhouse compartment planted with maize genotypes without any FAW infestation was included as control for each replication.

Foliar damage was assessed 1 week after infestation and, subsequently, weekly on five and four instances for the V5 and V7 treatments, respectively. Foliar damage score was not assessed for the infestation done at the R1 (silking) stage. At harvest, ears were handpicked from all plants in each plot. Ear damage, percentage ear rot, number of exit holes, and insect stem tunnel length were recorded. Foliar and ear damage was assessed using a scale of 1–9, where 1 = no visible damage and 9 = completely damaged (10) (score 1 to 2 is considered as resistant, 3 to 5 as tolerant and more than 5 as susceptible for foliar damage rating). Rotten percentage on the ear was assessed by determining the percent area of the ear affected by fungal molds (following FAW attack of the ear). The rotten percentage was evaluated by determining the percentage of each ear covered by symptoms using a class rating scale of 1 to 9, in which 1 = 0%, 2 = 1%–20%, 3 = 21%–30%, 4 = 31%–40%, 5 = 41%–50%, 6 = 51%–60%, 7 = 61%–70%, 8 = 71%–80%, 9 = 81%–100% of the kernels exhibiting visible symptoms of rotten grain. Grain weight was measured, and a sample of the grain was used to determine moisture content for each plot. Grain yield (t ha^−1^) was calculated from grain weight with adjustment for grain moisture content and shelling percentage. In addition, the percentage yield loss was calculated.

### Statistical analysis

Statistical analysis of life tables was performed using the Jackknife method as described by Hulting et al. (14). The pre-imaginal survivorship was calculated by dividing the number of individuals alive until adult emergence by the number of eggs laid by each cohort. The differences in intrinsic rate of increase (r_m_) values among populations reared on each maize genotype were calculated following the protocol described by Dixon (15). The intrinsic rate of increase is the number of births minus the number of deaths per generation time. Comparisons among the six hybrids were performed using Newman-Keuls sequential tests (16, 17) based on jackknife estimates of variance for r_m_ values (18). For any difference between two r_m_ from the sequence to be significant at the α level, the difference must be equal to or greater than


$$LSR\;=\;Q_{\propto \left[KV\right]}\;\sqrt{S^{2}av\;}\;\sqrt{\frac{n_{j}\;+\;n_{j}}{2n_{i}\;n_{j}}}$$

where K is the number of r_m_ values in the set whose range is tested, and V is the degrees of freedom. The n_i_ and n_j_ are the sample sizes of the r_m_ values, and Q_α[K,V]_ is a value from the table of the studentized range. S^2^_av_ is the weighted average variance of r_m_, and it is calculated as


$$S^{2}av\;=\;\frac{\Sigma^{\;a\;}\left(n_{i\;}\;-\;n_{j}\right)S^{2\;}\;i}{\Sigma^{a}\left(n_{i}\;-\;1\right)}$$

The sample size of the i^th^ r_m_ is n_i_, and S^2^_i_ is the jackknife estimate of the variance for the i^th^ r_m_.

Analysis of variance for different parameters recorded in laboratory and net house studies was performed with the PROC GLM and PROC GLIMMIX of SAS (19) for parametric and non-parametric data, respectively. Cohort was considered as a block effect in the model and each insect unit as a sampling error. The mean percentage data on hatchability, relative growth rate (RGR), absolute increment, pre-imaginal development time, adult longevity, and female fecundity were then subjected to the Tukey’s honest significantly difference (HSD) test. The leaf damage was analyzed using a repeated measures analysis consisting of two main effects: V5 and V7 infestation stages and FAW damage in 1, 2, 3, 4, and 5 weeks, respectively. The degree of freedom (df) was calculated using the Kenward and Roger II method (20). Ear damage, percentage rotten ear, number of stem exit holes, percentage of cumulative tunnel length, grain yield, and percentage yield loss were separated using HSD test at P = 0.05.

Percentage data (rotten, cumulative tunnel length) were arc sin–transformed, whereas count data were log-transformed before analysis although non-transformed results are presented in the tables and figures. Spearman correlation analysis was performed to determine the pairwise association among various damage parameters due to FAW and grain yield using PROC CORR of SAS (19). Stepwise regression was conducted to determine the effect of different FAW damage parameters on grain yield using PROC REG of SAS (19).

Because the growth rate did not differ significantly between males and females, the full data set was used in the analysis. The RGR (defined as relative logarithmic weight/time increase in insect biomass) was calculated for each genotype as follows (21):


RGR=  log(final weight /initial weight)/time

Absolute increment (AI, defined as the body mass growth rate relative to the initial body mass in time) was calculated as follows:


$$\text{AI }=\;\frac{\text{Final weight}-\text{Initial weight}}{\text{Time}}$$

Percentage yield loss was calculated for each genotype as follows:


$$\text{\% yield loss =}\frac{\text{Yield protected }-\text{ Yield infested}}{\text{Yield protected}}\;\;\times 100$$

where yield protected was the yield under non-infestation (control) and yield infested was the yield under FAW artificial infestation at V5, V7, and R1.

## Results

### Development time and longevity of FAW females

Maize genotype had a significant effect on larval development time, pre-imaginal development time, and the longevity of female FAW (**Table 1**). Larval development time was longest on the FAW-tolerant hybrid CKH191221 and shortest on FAW-susceptible OPV KDV4. Similarly, pre-imaginal development time was shortest on KDV4 and longest for larvae reared on the FAW-tolerant hybrids CKHFAW180294 and CKH191221 (**Table 1**). Female longevity was longer on CHK191221, CKHFAW180294, and KDV4 compared with ZM523.

**Table 1:** Mean (± S.E.) development time and female longevity of FAW larvae reared on leaf discs of six maize genotypes under controlled conditions at Katumani, Kenya, in 2019.

Maize type	Genotypes	Larval development time (in days)	Pre-imaginal development time (in days)	Female longevity (in days)
Tolerant	CKH191221	16.4 ± 0.44a	23.3 ± 0.20a	7.4 ± 0.24
	CKHFAW18029	15.7 ± 0.23b	23.4 ± 0.19a	7.3 ± 0.20
Commercial	WE2115	14.4 ± 0.20c	22.0 ± 0.18b	6.8 ± 0.24
	CKH10717	14.6 ± 0.12c	22.7 ± 0.26b	6.7 ± 0.22
OPV	ZM523	14.5 ± 0.16c	22.3 ± 0.23b	6.4 ± 0.16
	KDV4	13.7 ± 0.18d	21.0 ± 0.11c	7.2 ± 0.18
o	F-value	5.91	11.21	0.92
o	df	5,10	5,10	5,10
o	*P*	0.0085	0.0028	0.5087

Means within a column followed by different letters are significantly different according to Tukey’s HSD test at P ≤ 0.05.

### Relative growth rate, absolute increment, and fecundity of FAW

The RGR of FAW, absolute increment, fecundity, and egg hatchability differed significantly (*P* ≤ 0.0001) among the maize genotypes (**Table 2**). The lowest growth rate was observed for larvae reared on CKHFAW180294 and highest on OPVs, KDV4, and ZM523. The lowest and highest absolute increment was observed on the tolerant hybrid CKH191221 and OPV KDV4, respectively. Significantly more eggs were laid by FAW adults on the susceptible commercial check CKH10717 compared with adults on the tolerant hybrid CKH191221. Percent egg hatchability ranged from 55.2% for the tolerant hybrid CKHFAW180294 to 91.5% for KDV4 (**Table 2**).

**Table 2:** Mean (± S.E.) relative growth rate, absolute increment of FAW larvae, female fecundity, and percentage egg hatchability on six maize genotypes, at Katumani, Kenya, in 2019.

Maize type	Genotype	Relative growth rate at 12 days (day^−1^)	Absolute increment (mg day^−1^)	Female fecundity	Egg Hatchability (%)
Tolerant	CKH191221	0.185 ± 0.0028b	20.5 ± 0.46a	547.7 ± 67.43a	56.21 ± 5.85a
	CKHFAW180294	0.178 ± 0.0024a	22.2 ± 0.46b	769.5 ± 69.45b	55.15 ± 5.28a
Commercial	WE2115	0.199 ± 0.0027c	25.6 ± 0.52d	955.2 ± 60.01c	66.53 ± 5.48b
	CKH10717	0.194 ± 0.0029c	26.9 ± 0.48e	1136.3 ± 71.01d	82.17 ± 3.36c
OPV	ZM523	0.201 ± 0.0023d	23.7 ± 0.56c	748.0 ± 69.60b	70.87 ± 5.19b
	KDV4	0.201 ± 0.0023d	28.3 ± 0.47f	802.1 ± 85.55bc	91.52 ± 1.19d
	F-value df *P*	5.57 5,10 0.0104	12.46 5,10 0.0019	0.42 5,10 0.0043	4.14 5,10 0.0269

Means within a column followed by different letters are significantly different according to Tukey’s HSD test at P ≤ 0.05.

### Reproductive potential of FAW

There were significant differences among genotypes for intrinsic rates of increase (r_m_), net reproductive rate (R_o_), and mean generation time (G) (**Table 3**). A significantly lower r_m_ was observed for larvae reared on tolerant hybrid CKH191221 compared with the commercial checks (**Table 3**). A similar trend was observed for net reproductive rate (R_o_). Conversely, mean generation time (G) was higher for larvae reared on the tolerant hybrids, compared with the commercial hybrid and OPVs.

**Table 3:** Life table statistics of FAW reared on six maize genotypes at Katumani, Kenya, in 2019.

Maize type	Genotypes	Intrinsic rate of increase (r_m_)	Net reproductive rate (R_o_)	Mean generation time (G) (days)
Tolerant	CKH191221	0.24 ± 0.003a	218.12 ± 16.95a	22.8 ± 0.15a
	CKHFAW180294	0.26 ± 0.004b	302.91 ± 29.83b	21.6 ± 0.13b
Commercial	WE2115	0.35 ± 0.002d	997.53 ± 54.23d	19.5 ± 0.12d
	CKH10717	0.34 ± 0.002c	964.16 ± 36.70d	20.2 ± 0.12c
OPV	ZM523	0.36 ± 0.003e	851.19 ± 44.12c	18.7 ± 0.23e
	KDV4	0.41 ± 0.004f	1293.02 ± 75.87e	17.6 ± 0.11f
	df	42 to 58	41 to 56	44 to 58
	*P*	0.00014 to 0.21489	0.00001 to 0.61248	0.00010 to 0.00761

Means within a column followed by different letters are significantly different according to t test at P ≤ 0.05. P are the minimum and maximum p-values of the T-test among all pairwise tests. df are the minimum and maximum Df among all pairwise comparisons.

### Foliar damage under artificial FAW infestation at V5 and V7 stages under net house conditions

**Figure 1:**
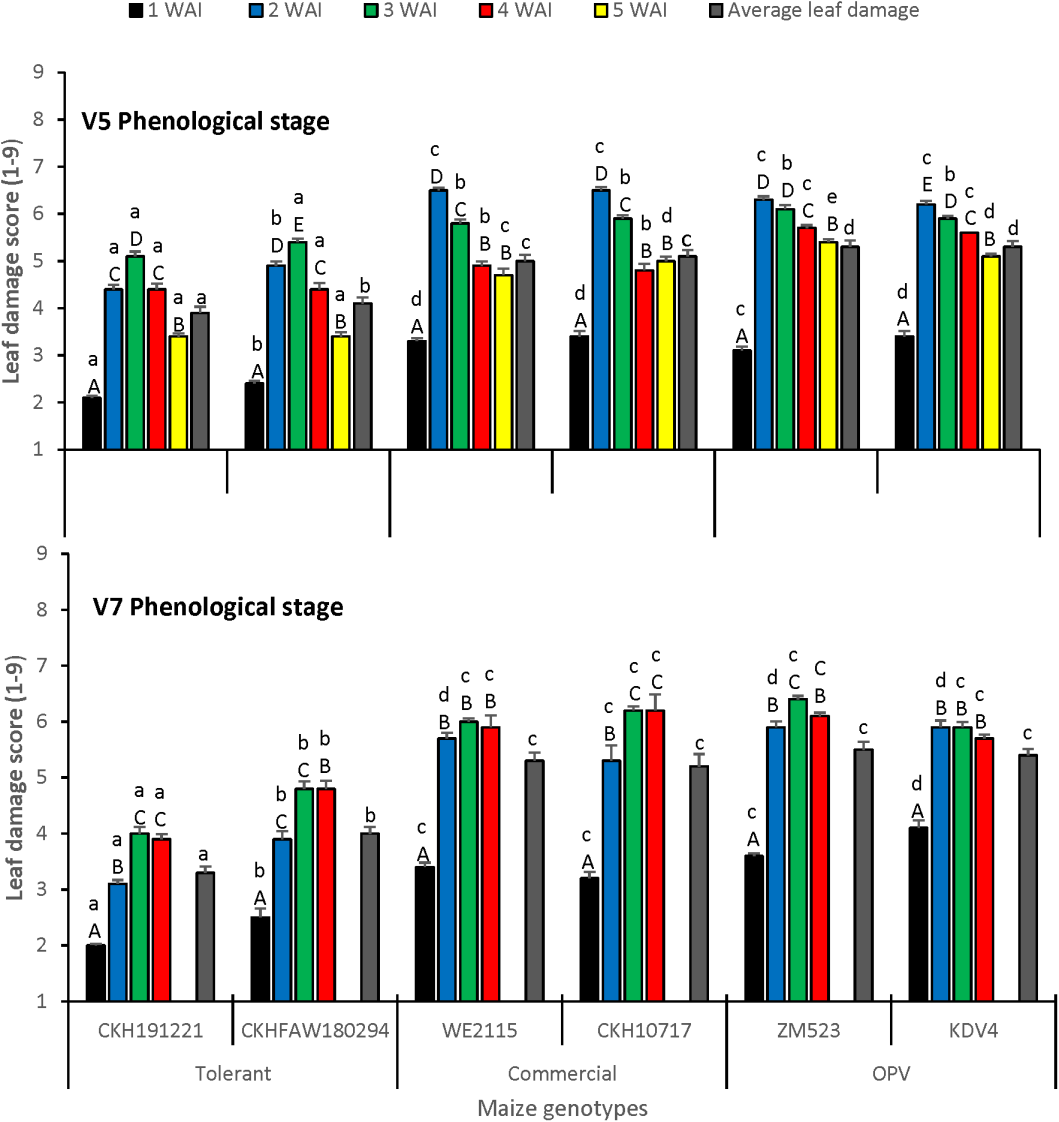
Leaf damage score on maize at V5 and V7 phenological stages under artificial infestation with FAW in an insect-proof net house at Kiboko, Kenya in 2019. Bars with the same lower-case letter(s) by genotypes and same capital letter in weeks are not signific antly different (SNK test; P < 0.05).

There was a significant difference in foliar damage among the scores taken at different periods (F = 346.25; df = 7,47.72; *P*< 0.0001) and among the genotypes tested (F = 11.69; df = 5,95.63; *P* = 0.0008). The interaction between the genotypes tested and the time of leaf damage score was significant (F = 2.49; df = 35,40.92; *P* = 0.0028) as well as the interaction between the genotypes tested and the infestation treatments (V5 and V7) (F = 3.65; df = 5,9.604; *P* = 0.0407). The lowest leaf damage was recorded in week 1 irrespective of treatment. The tolerant hybrid CKH191221 had the lowest leaf damage at V5 and V7 infestation stages at 1, 2, 3, and 5 weeks after infestation (**Figure 1**). Average leaf damage was lower for the tolerant hybrids and higher for the commercial checks and OPVs for both vegetative infestation stages.

### Ear damage due to artificial FAW infestation under net house conditions

Ear damage, percentage rotten ear, exit holes, tunnel length, and grain yield differed significantly among the maize genotypes at all three phenological stages (**Table 4**). Hybrid CKH191221 had the lowest ear damage for the three phenological stages (V5, V7, and R1), whereas the highest ear damage was recorded on ZM523 and WE2115 at V5 and V7 phenological stages. For infestation done at R1 stage, WE2115 showed the highest ear damage (F = 4.90; df = 15,36; *P<* 0.0001). Rotten ear percentage significantly differed between the hybrids and OPVs with the lowest percentage recorded on hybrids CKH191221 and CKHFAW180294 (F = 9.44; df = 3,36; *P<* 0.0001). The number of stem exit holes was higher on OPV KDV4 for infestation at all three phenological stages compared with CKH191221 (F = 2.00; df = 15,36; *P* = 0.0425). A similar trend was recorded with percentage tunnel length for all infestation treatments (F = 1.94; df = 15,36; *P* = 0.0493).

**Table 4:** Mean (± S. E.) ear damage, grain yield, and other parameters of six maize genotypes under artificial infestation with FAW at different phenological stages and control (no infestation) at Kiboko, Kenya, 2019.

Maize type	Genotype	Ear damage (1–9)	Rotten ear (%)	No. of stem exit holes (#)	Cumulative tunnel length (%)	Grain yield (tons ha^−1^)	% Yield loss
				**V5 stage**			
Tolerant	CKH191221	1.9 ± 0.05aB	2.73 ± 0.40aB	0.4 ± 0.03aB	0.82 ± 0.07aC	6.44 ± 0.11aC	35.41 ± 1.33bB
	CKHFAW180294	2.1 ± 0.03bB	3.03 ± 0.31aB	0.8 ± 0.01bB	1.60 ± 0.22bB	7.45 ± 0.35aB	25.35 ± 3.88aB
Commercial	WE2115	4.2 ± 0.17dB	36.46 ± 1.59bC	1.1 ± 0.13c	2.14 ± 0.28cB	5.46 ± 0.27bC	46.84 ± 3.32cB
	CKH10717	3.8 ± 0.12cB	36.59 ± 1.89bCc	1.3 ± 0.18cB	2.46 ± 0.40cC	6.96 ± 0.32aC	40.10 ± 4.35cB
OPV	ZM523	4.5 ± 0.19dB	41.44 ± 1.79cC	2.0 ± 0.63dB	4.14 ± 0.86dB	2.90 ± 0.19cC	55.66 ± 3.02dB
	KDV4	3.9 ± 0.14cB	37.22 ± 1.52bC	2.4 ± 0.02eB	5.25 ± 0.53dB	2.63 ± 0.08cD	54.34 ± 1.45dC
				V7 stage			
Tolerant	CKH191221	1.9 ± 0.03aB	2.41 ± 0.32aB	0.3 ± 0.03aB	0.49 ± 0.08aB	6.16 ± 0.11bC	38.21 ± 1.21bB
	CKHFAW180294	2.1 ± 0.04bB	3.95 ± 0.38aB	0.8 ± 0.09bB	1.75 ± 0.36bB	7.52 ± 0.34aB	24.65 ± 3.13aB
Commercial	WE2115	5.0 ± 0.17dC	46.97 ± 1.76cD	1.0 ± 0.14b	1.72 ± 0.33bA	5.72 ± 0.50cC	44.30 ± 5.43cB
	CKH10717	3.9 ± 0.08cB	38.84 ± 1.40bC	1.0 ± 0.13bB	1.80 ± 0.33bB	5.00 ± 0.18cD	56.97 ± 2.81dC
OPV	ZM523	5.2 ± 0.15dC	47.43 ± 1.92cC	1.2 ± 0.11bB	3.35 ± 0.41cB	2.34 ± 0.12eD	64.22 ± 1.75dC
	KDV4	4.0 ± 0.15cB	39.13 ± 1.35bC	2.1 ± 0.28cB	4.61 ± 0.68dB	3.12 ± 0.22dC	45.83 ± 3.15cB
				R1 stage			
Tolerant	CKH191221	1.6 ± 0.06aA	2.02 ± 0.33aB	0.2 ± 0.02aA	0.24 ± 0.04aA	8.88 ± 0.18bB	10.03 ± 1.97bA
	CKHFAW180294	1.9 ± 0.04bA	3.31 ± 0.27aB	0.3 ± 0.06aA	0.35 ± 0.08aA	9.39 ± 0.18aA	5.91 ± 1.37aA
Commercial	WE2115	3.5 ± 0.20eA	29.71 ± 2.74bB	0.9 ± 0.01d	1.27 ± 0.27cA	8.50 ± 0.30bB	17.23 ± 3.98cA
	CKH10717	2.8 ± 0.10cA	23.91 ± 1.91bB	0.7 ± 0.01cA	0.92 ± 0.01bA	9.21 ± 0.05aB	20.74 ± 1.56cA
OPV	ZM523	3.1 ± 0.22dA	27.01 ± 3.32bB	0.5 ± 0.01bA	0.81 ± 0.02bA	4.55 ± 0.20cB	30.43 ± 2.37dA
	KDV4	3.1 ± 0.08dA	32.28 ± 0.96cB	1.5 ± 0.14eA	2.40 ± 0.22dA	4.34 ± 0.12cB	24.65 ± 2.77cA
				Control		
Tolerant	CKH191221		1.30 ± 0.25A			9.97 ± 0.04bA	
	CKHFAW180294		1.54 ± 0.13A			9.98 ± 0.13bA	
Commercial	WE2115		1.04 ± 0.24A			10.27 ± 0.17bA	
	CKH10717		1.22 ± 0.25A			11.62 ± 0.27aA	
OPV	ZM523		1.80 ± 0.24A			6.54 ± 0.31cA	
	KDV4		1.54 ± 0.24A			5.76 ± 0.09dA	
	F-value df *P*	6.33 15,40 ≤0.0001	11.19 15,40 ≤0.0001	1.45 15,40 ≤0.0001	4.15 15,40 ≤0.0001	9.78 15,40 ≤0.0001	0.68 10,30 0.0383

Means within column followed by the same lower-case letter(s), and within column and phenological stage followed by the same capital letter(s) are not significantly different according to LSD test; P< 0.05.

Grain yield varied significantly among genotypes for all treatments (**Table 4**) (F = 10.34; df = 15,36; *P<* 0.0001). Grain yield was significantly higher in the non-infested controls compared with the infested plots. Compared with the corresponding controls, grain yield was significantly reduced in the commercial check hybrid WE2115 by 46.8% and 56.9% at V5 and V7 phenological stages, respectively. Similarly, there was 64.2% yield reduction on the OPV ZM523 at V7 stage. However, on the FAW-tolerant hybrids, yield reductions were significantly lower: 38% at V7 stage for CKH191221 and 25% at V5 for CKHFAW180294 (**Table 4**) (F = 0.92; df = 10,24; *P* = 0.0333).

### Relationship between FAW damage parameters and maize grain yield

For quantitative and statistical analysis of the effect of FAW damage to maize grain yields, spearman correlation and stepwise regressions were used with grain yields in each phenological stage as a dependent variable and leaf damage (weeks 1–5), ear damage, rotten ear percentage, number stem exit holes, and percentage cumulative tunnel length as independent variables.

The correlations between grain yield and FAW damage parameters exhibited significant association among the test genotypes. Grain yield was significantly and negatively correlated with leaf damage at V5 (−0.505 on WE2115, −0.952 on CKH10717, and −0.735 for ZM523) and and V7 (−0.799, −0.657, −0.804, and −0.574 for CHKFAW190284, WE2115, ZM523, and KDV4, respectively). Similarly, grain yield was significantly and negatively correlated with ear damage at V5 (−0.754 for WE2115 and −0.739 for ZM523), V7 (−0.549, −0.928, and −0.663 for CHK191221, WE2115, and ZM523, respectively), and R1 (−0.603, −0.933, and −0.584 for CKH10717, ZM523, and KDV4, respectively). The number of exit holes was negatively correlated with yield grain at V5 (−0.506, −0.614, −0.855, −0.695, −0.609, and −0.573 for CHK191221, CHKFAW190284, WE2115, CHK10717, ZM523, and KDV4, respectively), V7 (−0.873 for WE2115), and R1 (−0.768, −0.780, −0.731, −0.843, and −0.547 for CHK191221, WE2115, CHK10717, ZM523, and KDV4, respectively). Grain yield was significantly and negatively correlated with the percentage of rotten ear [phenological stages V5 (−0.559, −0.625, −0.659, and −0.504 for WE2115, CHK10171, ZM523, and KDV4, respectively), V7 (−0.873 for WE2115), and R1 (−0.508, −0.487, and −0.931 for CHKFAW190284, WE2115, and ZM523, respectively)]. The percentage cumulative tunnel length was negatively correlated to the grain yield among tested genotypes at V5 (−0.649, −0.830, −0.875, −0.762, and −0.515 for CHKFAW190284, WE2115, CHK10717, ZM523, and KDV4, respectively), V7 (−0.577, −0.787, −0.566, −0.610, −0.732, and −0.851 for CHK191221, CHKFAW190284, WE2115, CHK10717, ZM523, and KDV4, respectively), and R1 (−0.528, −0.839, −0.530, and −0.928 for CHK191221, CHKFAW190284, WE2115, and ZM523, respectively).

For all three infested treatments at different phenological stages (V5, V7, and R1), stepwise regressions showed that leaf damage across 5 weeks negatively affected grain yields (**Table 5**). Across all genotypes and among all phenological stages, ear damage significantly affected grain yield.

**Table 5:** Stepwise regression analysis between FAW damage parameters and grain yield of six maize genotypes artificially infested with FAW at V5, V7, and R1 stages (n = 432).

			Leaf damage (weeks)					Ear damage	Rotten ears %	No. of exit holes	% Tunnel length	P-Value	R^2^
		Intercept	1	2	3	4	5					
Maize type	Genotype	Grain Yield											
									**V5**				
Tolerant	CKH191221	1.876	−1.83	−2.21	−1.61			−0.678				0.0001	0.9274
	CKHFAW180294	1.195		−4.81	−5.46			−1.551			−0.053	0.0001	0.9350
Commercial	WE2115	0.054			−0.86	−1.44		−0.919				0.0001	0.7582
	CKH10717	7.675		−5.40		−2.57		−1.248			−0.066	0.0001	0.9151
OPV	ZM523	1.483	−0.75	−1.90	−1.70	−2.31		−1.161				0.0001	0.9187
	KDV4	0.192	−1.04				−1.88	−0.113	−0.004			0.0047	0.6612
			V7										
Tolerant	CKH191221	0.473		−1.83		−0.64	−0.39	−0.769	−0.003	−0.010		0.0001	0.8984
	CKHFAW180294	0.578		−2.75	−1.44			−1.948	−0.040			0.0002	0.8544
Commercial	WE2115	1.068		−1.24				−3.000	−0.010			0.0001	0.9218
	CKH10717	1.633			−2.81	−3.54						0.0001	0.7365
OPV	ZM523	10.55		−2.11		−1.32	−2.12	−0.342			−0.045	0.0006	0.8041
	KDV4	0.298			−1.08			−0.010	−0.004			0.0001	0.7935
			R1										
Tolerant	CKH191221	0.809						−0.951			−0.890	0.0004	0.6459
	CKHFAW180294	0.365						−0.067				0.0002	0.5491
Commercial	WE2115	9.091						−1.673		−3.453	−1.950	0.0018	0.6451
	CKH10717	2.957						−3.497				0.0008	0.5176
OPV	ZM523	2.519						−0.551	−0.043			0.0008	0.6162
	KDV4	0.365						−1.994				0.0079	0.6355

## Discussion

This study showed that the FAW-tolerant hybrids exhibited longer pre-imaginal development time in FAW than susceptible genotypes and, in the case of CKH191221, a significantly lower female fecundity. Development time recorded in this study was lower by 50% to 60% compared with that reported on maize infested by FAW in the United States (22). Tendeng et al. (23) demonstrated that the total duration of FAW reared on maize was between 22 and 28 days at 25°C, with FAW producing an average of 15 generations per year. In this study, a similar trend was observed though with a longer developmental time on the FAW-tolerant hybrids compared with the susceptible genotypes. Previous studies on larval developmental time in maize reported 11 days (24), 21 days (25), and 14 days (23) at 25°C. These differences larval developmental period can be explained by the environmental conditions and the larval capacity to consume the host plant as well as the experimental setup (26, 27).

The two FAW-tolerant genotypes showed the lowest RGRs and absolute increments, indicating an antibiosis effect. Similarly, Lima et al. (28) and de Paiva et al. (29) reported antibiosis for FAW as shown by a low larval viability, small larval weight, short adult longevity, and low emergence rates. Genotypes CKH191221 and CKHFAW180294 had the least number of eggs laid. Female fecundity recorded in this study was similar to that reported in several studies (25, 30, 31) but is lower than those reported by Kumela et al. (32) and Tendeng et al. (23). The low female fecundity observed on the tolerant maize genotypes could be attributed to components of host plant quality such as carbon, nitrogen, and defensive metabolites, which directly affect the fecundity (33).

Differences in life table parameters like in developmental time, survival rate, and fecundity of FAW reared on the different genotypes might be explained by allelochemicals present in the leave such as alkaloids, glucosinolates, protein inhibitors, and lipids (34, 35). The mean generation times (G) recorded in this study were 9% to 40% shorter than those reported by Pinto et al. (36) on five maize genotypes. Pinto et al. (36) reported a R_o_ of 755 to 920, which was in the same range as that obtained on the susceptible genotypes. The number of eggs laid by an insect is usually determined at oogenesis, in which physiological processes are determined by availability of nutrients in the female’s body (37). This is mainly caused by the adequate food ingested and assimilated during larval development (36, 38). The performance of FAW on the susceptible hybrids and OPVs in this study may be related to the higher concentration of nutrients such as proteins and amino acids in the food resource as well the ease of digestibility (35). Conversely, the tolerant hybrids appeared to have factors that inhibit the growth of FAW, resulting into lower fecundity.

Under artificial infestation, FAW-tolerant hybrids showed lower damage at V5 and V7 phenological stages. Maize infested early suffered more damage compared with that infested at a later phenological stage (39). On older leaves, foliar damage was reduced, which is attributed to leaf tissues being tough and indigestible, thereby limiting FAW feeding and growth (40). A significant negative relationship between grain yield and FAW damage was observed at V5 and V7 phenological stages, implying that leaf damage at V5 and V7 significantly affected yields of all the tested genotypes. A study by Thomison et al. (41) estimated that 70% defoliation at the 12-leaf stage would result in a 15% yield reduction, whereas 25% defoliation never caused more than 9% yield loss and 50% defoliation at 18-leaf stage the damage caused less than 5% yield loss.

Under high infestation pressure, some larvae evade cannibalism by entering the stem resulting tunneling and exit holes (8, 42). Tunnel length was shorter in the tolerant than susceptible hybrids and OPVs. Tunneling leads to reduced movement of nutrient in the plants. Thus, tunnel length and number of stem exit holes can be translated into grain yield loss (43–45) and can be used as parameters to assess severity of FAW infestation. Similar to the findings by Nwanze et al. (46), grain yield was negatively correlated with number of exit holes and cumulative tunnel length in this study.

The effect on grain yields due to FAW infestation across all the phenological stages differed significantly among genotypes. Higher yield loss was recorded for infestation treatments V5 and V7 as compared with R1. When the infestation is done at the vegetative stage, larvae move to the whorl, thereafter to the emerging tassel, and then to the developing ear (40). The larvae first feed on the husks before reaching the developing kernels after penetrating the ear through the tip (47, 48). A yield reduction of 15% when 98% of plants were artificially infested by FAW at 8- to 10-leaf stage was reported by Cruz and Turpin (8). These authors also reported 18% yield reduction with 31% FAW infestation. This is in line with studies by Hruska and Gladstone (49), who carried out a study with 100% infestation of the plants and recorded a 34% yield reduction by FAW. In early stages of crop development FAW, attacks maize extensively causing a lot of defoliation. In case of attack during the late stages of crop growth, FAW larvae may cause lesser damage to the grain yield compared with early attack. The results indicate that the larval stage at the early phenological stages (V5 and V7) might be the most appropriate time to target management options because a much higher population suppression is achievable with small increment of mortality (50) and for germplasm screening.

## Conclusion

The FAW-tolerant experimental hybrids led to lower FAW growth rates and fecundity in laboratory studies and showed lower insect damage parameters and grain yield loss, as compared with the susceptible genotypes in net house experiments. This study should serve as a baseline for further studies on resistance to FAW in tropical maize germplasm and validation of resistance in other sources of maize germplasm. Laboratory assays and net house trials do not necessarily reflect the exact conditions encountered under field conditions. Therefore, it is important to screen the genotypes under field conditions to ascertain their performance under natural FAW pressure in the target agroecological conditions.

## Data availability statement

The original contributions presented in the study are included in the article/supplementary material. Further inquiries can be directed to the corresponding author.

## Author contributions

Conceptualization: GNA, AYB, MA, BMP, DM, and YB. Methodology: GNA, AYB, RK-G, and SEN, MA, DM, and YB. Software: GNA and AYB. Validation: AYB, RK-G, and SEN. Formal analysis: GNA and AYB. Data curation: GNA and AYB. Writing—original draft preparation: GNA. Reviewing: AYB, RK-G, DM, YB, MA, SEN, and BMP. Project administration: BMP and MA. Funding acquisition: BMP and AB. All authors contributed to the article and approved the submitted version.

## Funding

The study was undertaken through the financial support from the United States Agency for International Development (USAID) Bureau for Resilience and Food Security under Grant # BFS-IO-17-00005 as part of Feed the Future activity, under the Fall Armyworm Management Project to CIMMYT, and the CGIAR Research Program on Maize (MAIZE) Windows 1 and 2. Any opinions, findings, conclusions, or recommendations expressed here are those of the authors alone.

## Acknowledgments

The authors are grateful for the collaboration extended by the Kenya Agricultural and Livestock Research Organization (KALRO) Research Centers at Machakos and Kiboko and to the Department of Agriculture, Science and Technology, Kenyatta University, for necessary support. Special thanks to CIMMYT Technicians Charles Marangu and Gerphas Ogola for their technical support for this study.

## Conflict of interest

Author MA is employed by Amobant LLC but previously worked at CIMMYT.

The remaining authors declare that the research was conducted in the absence of any commercial or financial relationships that could be construed as a potential conflict of interest.

## Publisher’s note

All claims expressed in this article are solely those of the authors and do not necessarily represent those of their affiliated organizations, or those of the publisher, the editors and the reviewers. Any product that may be evaluated in this article, or claim that may be made by its manufacturer, is not guaranteed or endorsed by the publisher.
